# Frequency‐dependent resemblance of male‐colored females to males in a damselfly

**DOI:** 10.1111/1744-7917.12584

**Published:** 2018-04-06

**Authors:** Wicher Vos, Jan Komdeur, Martijn Hammers

**Affiliations:** ^1^ Behavioural and Physiological Ecology, Groningen Institute for Evolutionary Life Sciences University of Groningen Groningen The Netherlands

Dear editor,

Mimetic protection is most effective when mimics are relatively rare (Pfennig *et al*., 2001). In polymorphic damselfly species, male‐colored female morphs may avoid costly male mating attempts because they are not immediately recognized as a suitable mating partner (van Gossum *et al*., 2008). We investigated morphological resemblance of male‐colored females to males across six populations of the polymorphic blue‐tailed damselfly *Ischnura elegans* (Vander Linden). We found that male‐colored females resembled males more closely with an increasing ratio of male‐colored females to other female morphs. Our results suggest that the degree of mimetic fidelity is frequency‐dependent.

Genetically determined color polymorphisms have evolved throughout the tree of life and studying them helps in understanding the selective forces that affect the maintenance of genetic diversity (Gray & McKinnon, 2007). In some species, color polymorphism is limited to one sex. Such sex‐limited color polymorphisms has probably evolved in response to sex‐specific predation, sexual competition, or sexual conflict (Stamps & Gon III, 1983; Gross, 1996; Svensson *et al*., 2009).

In *I. elegans*, mature males occur in one color (blue) and mature females occur in three genetically controlled color morphs (Sánchez‐Guillén *et al*., 2005): *infuscans* (olive green), *rufescens‐obsoleta* (brown‐red) and androchrome (“male‐colored”: blue or green‐blue). In this species, females suffer from excessive male mating harassment, which is costly in terms of female fitness (Gosden & Svensson, 2009). Female morph frequencies differ greatly between populations (Cordero‐Rivera & Sánchez‐Guillén, 2007) and frequency‐dependent male mating decisions (Fincke, 2004) lead to a selective advantage for the rarer female morph(s) due to reduced male harassment (van Gossum *et al*., 2001; Svensson *et al*., 2005). Independent of their abundance in a population, androchrome females generally experience lower levels of male harassment than other female morphs (Hammers & van Gossum, 2008). Indeed, in damselflies of the genus *Ischnura*, androchrome females are likely functional male‐mimics (Robertson, 1985) that avoid male harassment due to their similarity to conspecific males in terms of coloration (van Gossum *et al*., 2011), behavior (van Gossum *et al*., 2001), and body size and shape (Abbott & Gosden, 2009).

Mimicry theory predicts that the effectiveness of mimetic protection is frequency‐dependent; mimetic protection is predicted to break down when mimics (androchromes) become more abundant relative to their models (males), or relative to alternative “prey” (other female morphs) (Hetz & Slobodchikoff, 1988; Harper & Pfennig, 2007; Iserbyt *et al*., 2011). Therefore, with an increase in (i) the ratio of androchromes to males and (ii) the ratio of androchrome females to other female morphs (i.e., alternative mating partners), it is predicted that androchromes should resemble males closer to maintain the efficiency of mimetic protection, whereas this is not expected for the other female morphs (Iserbyt *et al*., 2011). In this study, we investigated these predictions in six populations of the damselfly *I. elegans*.

Data were collected in six populations in the Netherlands during one visit per population between June and August 2016 (Table 1). Estimates of female morph frequency and sex ratio (Table 1) were obtained by “sweep‐netting” through shoreline vegetation between 08.00 h and 10.00 h (Hammers & van Gossum, 2008). Across populations, the ratio of androchrome females to males ranged from 0.12 to 0.79 (mean ± SE = 0.40 ± 0.12) and the ratio of androchrome females to other female morphs ranged from 0.57 to 5.50 (mean ± SE = 2.89 ± 0.74; Table 1). In addition, we collected 262 individuals from the six populations (85 males, 79 androchromes, 38 *infuscans*, and 60 *rufescens‐obsoleta*) for morphological measurements (Table 1). These collected damselflies were immediately killed and preserved in 97% ethanol. In order to measure morphology, individuals were placed on blotting paper for 2 min to allow for standardized absorption and evaporation of the ethanol (Iserbyt *et al*., 2011), and weighed on a digital scale to the nearest 0.1 mg. Then, the right hind wing was removed and the individual was positioned laterally on graph paper (0.5 cm grid), together with its right hind wing and photographed at a distance of *ca* 15 cm. Following Iserbyt *et al*. (2011), we used these pictures to measure body length, length of the fourth abdominal segment (hereafter: S4), width of S4, wing length and wing surface (Fig. 1), using the program ImageJ (NIH, Bethesda). Previous studies showed that morphological measures are often heritable and may differ between female color morphs and males in *I. elegans* (Abbott & Gosden, 2009; Abbott & Svensson, 2010). As several of these measurements are correlated, we performed a principal component analysis with varimax rotation and extracted two principal components, which explained 84% of the variance in the six measurements (PC1: 61%, PC2: 23%). PC1 included measurements that were associated with overall size and abdomen width, and PC2 included measures associated with body length (Table S1). From a mimicry perspective, the width of S4 may be particularly relevant as S4 width in androchromes is intermediate between smaller males and the other two female morphs, but positively associated with fecundity (Gosden & Svensson, 2009). Therefore, we also considered S4 width separately. Following Iserbyt *et al*. (2011), we also calculated two aspects of maneuverability: “aspect ratio” (wing length^2^/wing surface) and “wing load” (mass/[4 × wing surface]).

**Table 1:** Sampling locations in the Netherlands and numbers of mature males and female morphs of *Ischnura elegans* used for estimating morph frequencies and sex ratio. The numbers between brackets are the number of individuals collected and used for morphological measurements. PC1 and PC2 are the mean principal component scores for males in each population

Location	Sampling date	Latitude	Longitude	Males	Andro	*infusc*	*ruf‐obs*	PC1—size (males)	PC2—length (males)
Vinkhuizen	06‐Jun‐2016	53°13′48.6"	6°31′09.4"	14 (15)	11 (10)	1 (5)	1 (10)	−0.98	0.32
Monster	08‐Jun‐2016	52°00′35.1"	4°10′52.3"	112 (15)	13 (15)	10 (15)	13 (15)	−0.73	0.41
Maasvlakte	09‐Jun‐2016	51°56′07.8"	4°05′26.2"	33 (10)	10 (10)	2 (3)	5 (5)	−0.75	0.36
Helpman	13‐Jun‐2016	53°11′46.4"	6°34′31.4"	94 (15)	26 (15)	3 (8)	4 (14)	−0.93	0.14
Lettelbert	23‐Jul‐2016	53°11′36.6"	6°24′55.8"	16 (15)	12 (14)	3 (5)	1 (4)	−1.33	0.51
Eelde	05‐Aug‐2016	53°08′01.0"	6°32′33.9"	55 (15)	10 (15)	2 (2)	2 (12)	−1.55	−0.62

Andro = androchome females; *infusc = infuscans* females; *ruf‐obs* = *rufescens‐obsoleta* females.

**Figure 1:**
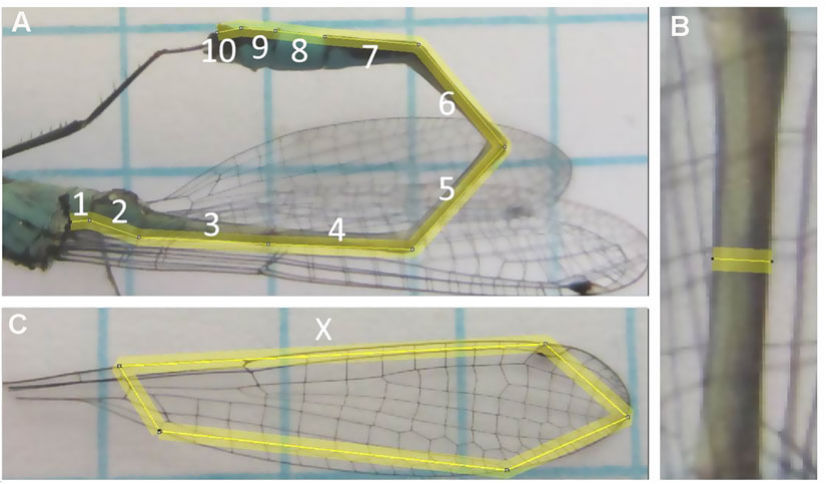
Example of morphological measurements. (A) The measurement of the length of the abdomen (numbers refer to the different abdominal segments). (B) The measurement of the width of abdominal segment S4. (C) The measurement of wing length (indicated with X) and wing surface.

To assess whether and how the size and shape of female morphs and males varies within and across populations we performed General Linear Models with (i) PC1, (ii) PC2, (iii) S4 width, (iv) aspect ratio, and (v) wing load as the dependent variables, and with population (6‐class factor) and morph (4‐class factor) as predictors. PC1, PC2, and S4 width differed significantly between morphs and between populations, but aspect ratio and wing load did not differ between morphs (Table 2; Fig. S1). As expected, PC1 and S4 width of androchromes was intermediate between males and the other two female morphs, but this was not the case for PC2 (Fig. S1). For PC1, S4 width and PC2, the interaction between morph and population was significant (Table 2), suggesting that the size differences between morphs vary across populations.

**Table 2:** Results of general linear models investigating whether principal component scores (PC1 and PC2), abdomen width (S4), aspect ratio and wing load differed between morphs and across populations of *Ischnura elegans*. The significance of the main effects was determined after dropping the interaction term from the model. Significant effects are in bold

	Morph		Population		Morph × population	
	*F*_(3,253)_	*P*	*F*_(5,253)_	*P*	*F*_(15,238)_	*P*
PC1 (size)	**246.14**	**<0.001**	**42.15**	**<0.001**	**1.78**	**0.038**
PC2 (length)	**3.46**	**0.017**	**19.51**	**<0.001**	**2.08**	**0.012**
S4 width	**135.74**	**<0.001**	**12.30**	**<0.001**	**3.06**	**<0.001**
Aspect ratio	1.12	0.344	**3.38**	**0.006**	0.59	0.880
Wing load	1.16	0.327	**8.10**	**<0.001**	1.18	0.287

We then tested whether varying size differences between female morphs and males across populations (for PC1, PC2, and S4 width) could be explained by the ratio of androchromes to males or to the other (nonmimetic) female morphs. For each population, this size difference was calculated as the mean value for the female morph minus the mean value for males. Contrary to expectation, we found no evidence for an increased resemblance of androchrome females to males with an increase in the ratio of androchrome females to males (PC1: *r* = −0.47, *P* = 0.350; PC2: *r* = −0.54, *P* = 0.265; S4 width: *r* = −0.42, *P =* 0.404). A study on the female polymorphic sedge sprite *Nehalennia irene* (Hagen) showed that the similarity of androchrome females to males increased with an increase in the ratio of androchrome females to males (Iserbyt *et al*., 2011). Although the benefits and costs of being an andromorph likely depend on their frequency relative to both males and other female morphs (Fincke, 2004), a possible explanation for the difference between these studies is that the relative influence of these two variables may differ between locations and species.

We found that size differences between androchromes and males declined significantly with an increase in the ratio of androchrome females to other female morphs in the population (Figs. 2 and S2; PC1: *r* = −0.81, *P* = 0.050; S4 width: *r* = −0.85, *P* = 0.034). These associations were not significant for the other female morphs or for PC2 (all *P >* 0.277). Although these results suggest that androchrome size becomes more male‐like in populations where androchromes are relatively common compared to the other female morphs, the results should be interpreted with caution. First, there is considerable uncertainty in our estimates of the ratio of androchromes to other female morphs as a relatively low number of mature females were available to estimate population morph frequencies (mean ± SE = 21.50 ± 4.17, range 13–36). Second, other factors that influence size might generate a pattern similar to what we found in our study. For example, the two populations furthest to the south had the lowest ratio of androchromes to other female morphs, the largest individuals, and the largest size difference between androchrome females and males. If constraints on male size would be larger than constraints on female size, this might provide an alternative explanation for the size decline. However, the finding that the similarity of androchrome females to males, but not the similarity of the other two female morphs to males, increases with the ratio of androchromes to other female morphs suggests that that size similarity is shaped by frequency‐dependent mimicry rather than by size differences *per se*.

**Figure 2:**
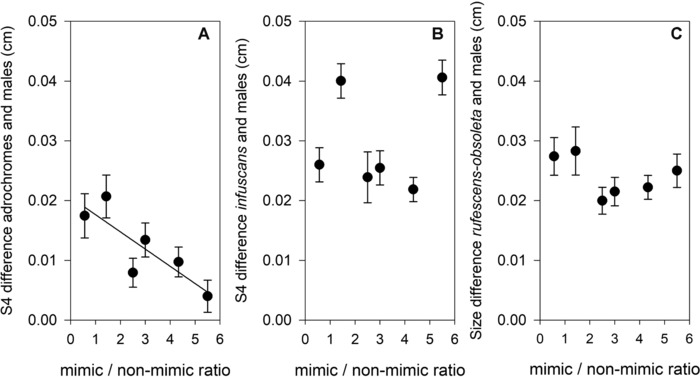
The size difference (± SE) in the width of the fourth abdominal segment (S4) between (A) androchrome, (B) *infuscans*, and (C) *rufescens‐obsoleta* (right panel) female morphs and males in relation to the ratio of androchrome females to the other female morphs (mimic/nonmimic ratio).

The observation that the size differences between androchromes and males decline when androchromes are relatively abundant, also suggests a trade‐of between fecundity and mimetic fidelity with respect to body size. The intensity of male harassment may affect the balance of this trade‐off (Gosden & Svensson, 2009), ultimately selecting for increased similarity of androchromes to males with increasing harassment levels. Therefore, the smaller size of androchrome females relative to the two nonmimetic female morphs, and the increased similarity to males in populations with a high ratio of androchromes to other female morphs may have resulted from selection to maintain the efficiency of mimetic protection.

## Disclosure

We have no conflicts of interest.

## Supporting information


**Fig. S1**. Morphological differences between males and the three female morphs for (A) PC1, (B) PC2, (C) S4 width, (D) aspect ratio, and (E) wing load. Data are means ± SE. Significances of pairwise contrasts (Tukey's HSD) are indicated: *P < 0.05; ***P < 0.001.
**Fig. S2**. The size difference ± SE (PC1—in gray) between (A) androchrome, (B) *infuscans*, and (C) *rufescens‐obsoleta* female morphs and males in relation to the ratio of androchrome females to the other female morphs (mimic/nonmimic ratio). The mean ± SE PC1 values are also indicated. The open dots are PC1 values for males and are identical in the three panels. The closed black dots are PC1 values for each female morph.
**Table S1**. Correlations between the six traits measured and the two principal components. Correlations >0.5 are in bold.
