# Supplementary material for: Frequency‐dependent resemblance of male‐colored females to males in a damselfly
Source: Insect Sci. 2018 Apr 6;26(5):958–62. doi: 10.1111/1744-7917.12584 (PMC7379685; doi:10.1111/1744-7917.12584)
Supplement: Supplementary file 1 — Fig. S1. Morphological differences between males and the three female morphs for (A) PC1, (B) PC2, (C) S4 width, (D) aspect ratio, and (E) wing load. Data are means ± SE. Significances of pairwise contrasts (Tukey's HSD) are indicated: *P < 0.05; ***P < 0.001. Fig. S2. The size difference ± SE (PC1—in gray) between (A) androchrome, (B) infuscans, and (C) rufescens‐obsoleta female morphs and males in relation to the ratio of androchrome females to the other female morphs (mimic/nonmimic ratio). The mean ± SE PC1 values are also indicated. The open dots are PC1 values for males and are identical in the three panels. The closed black dots are PC1 values for each female morph. Table S1. Correlations between the six traits measured and the two principal components. Correlations >0.5 are in bold. [file INS-26-958-s001.docx]

SUPPLEMENTARY MATERIALS

**Figure S1**

Morphological differences between males and the three female morphs for (A) PC1, (B) PC2, (C) S4 width, (D) aspect ratio and (E) wing load. Data are means ± SE. Significances of pairwise contrasts (Tukey HSD) are indicated: * = *P* < 0.05; *** *P* < 0.001.

**
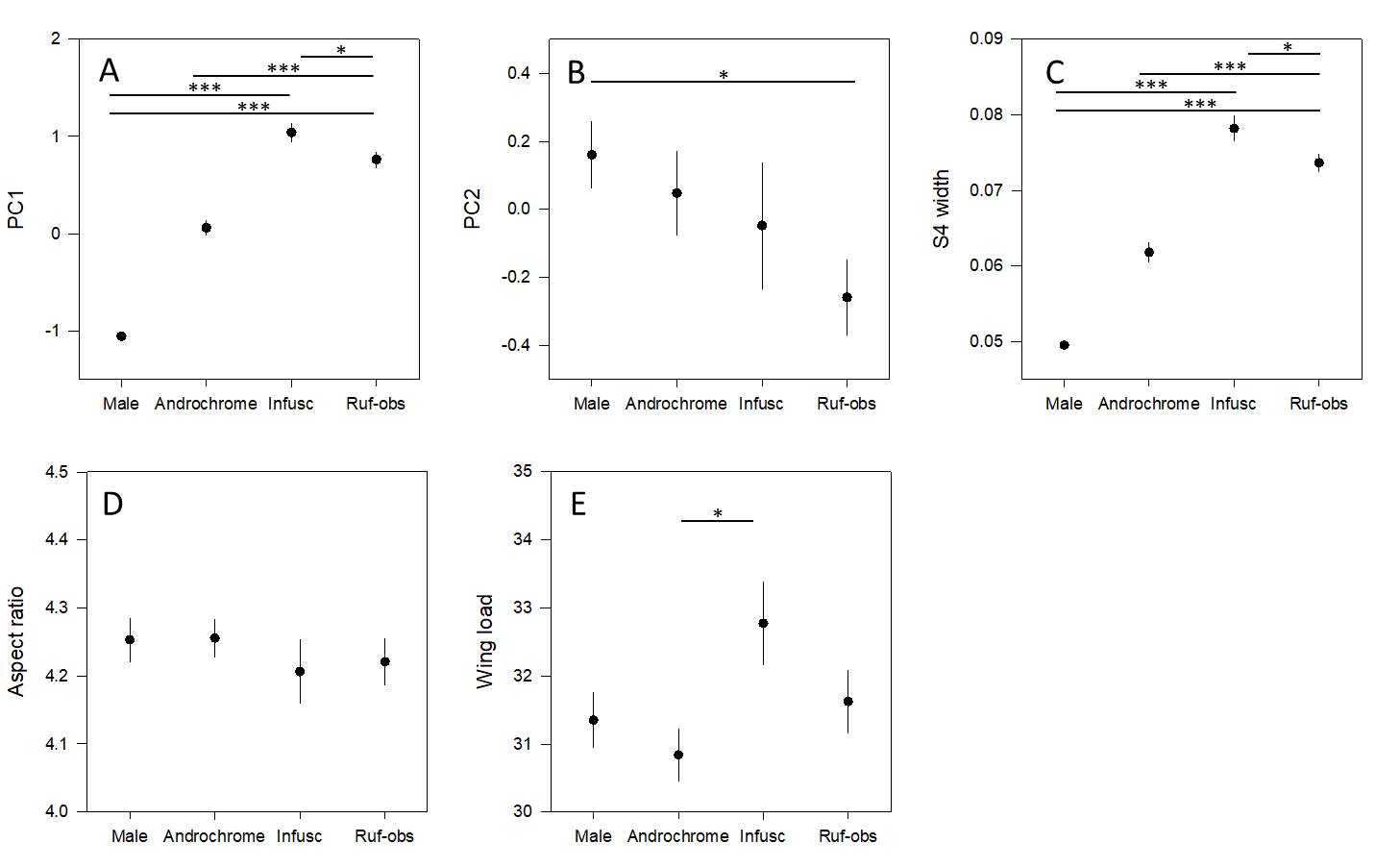
**

**Figure S2**

The size difference ± SE in size (PC1 – in gray) between (A) androchrome, (B) *infuscans* and (C) *rufescens-obsoleta* female morphs and males in relation to the ratio of androchrome females to the other female morphs (mimic/non-mimic ratio). The mean ± SE PC1 values are also indicated. The open dots are PC1 values for males and are identical in the three panels. The closed black dots are PC1 values for each female morph.

**
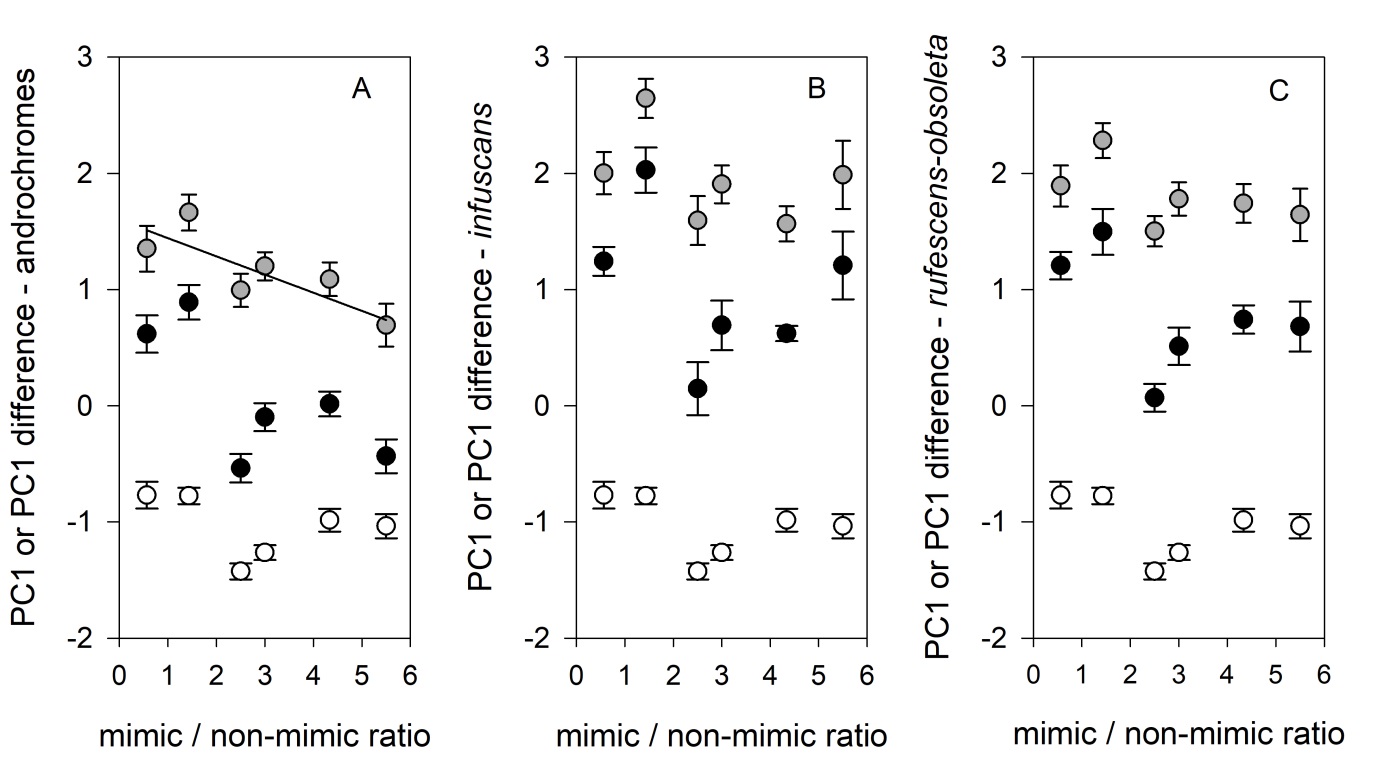
**

**Table S1**

Correlations between the six traits measured and the three principal components. Correlations > 0.5 are in bold.

|  |  |  |
| --- | --- | --- |
|  |  |  |
| Variable | PC1 | PC2 |
| Abdomen length | 0.23 | **0.89** |
| Segment S4 length | 0.08 | **0.91** |
| Segment S4 width | **0.86** | -0.07 |
| Wing length | **0.90** | 0.23 |
| Wing surface | **0.91** | 0.25 |
| Body mass | **0.86** | 0.31 |
